# Multi-target neuroprotection of carbon dots derived from Crinis Carbonisatus in multiple acute epilepsy model

**DOI:** 10.3389/fmolb.2026.1772499

**Published:** 2026-02-25

**Authors:** Kai Cheng, Jie Hu, Xiaoke Wang, Yifan Zhang, Xinrong Tian, Yan Huang, Chenxin He, Xiwen Zhang, Peng Zou, Jinyu Ma, Xiaohan Qu, Yue Zhang, Hui Kong, Yan Zhao

**Affiliations:** 1 School of Traditional Chinese Medicine, Beijing University of Chinese Medicine, Beijing, China; 2 Dongzhimen Hospital, Beijing University of Chinese Medicine, Beijing, China; 3 Encephalopathy Hospital, The First Affiliated Hospital of Henan University of Chinese Medicine, Zhengzhou, China; 4 School of Life Sciences, Beijing University of Chinese Medicine, Beijing, China

**Keywords:** carbon dots, Crinis Carbonisatus, epilepsy, neuroinflammation, neuroprotection

## Abstract

**Background:**

Epilepsy remains a prevalent neurological disorder characterized by spontaneous, recurrent seizures. Despite available treatments, there is a critical lack of safe and effective strategies for long-term management and control. Crinis Carbonisatus (CrCi), the carbonized product of human hair, has been utilized for millennia to manage epilepsy and hemorrhagic disorders in Traditional Chinese Medicine. In our previous work, we successfully isolated Carbon Dots (CrCi-CDs) from CrCi and demonstrated their neuroprotective activity against ischemic stroke. Extending this rationale to seizure management, the present study investigates the potential antiepileptic efficacy of CrCi-CDs in acute epilepsy models.

**Methods:**

CrCi-CDs were synthesized via the calcination of human hair at 350 °C, followed by aqueous extraction and purification. To evaluate their antiepileptic potential, acute epilepsy models were established in mice using three distinct chemoconvulsants: Pentylenetetrazole (PTZ), Pilocarpine (PILO), and Penicillin (PNC). We systematically assessed the ability of CrCi-CDs to attenuate seizure severity by modulating neuronal excitability, suppressing neuroinflammation, and mitigating oxidative stress. Furthermore, the PTZ-induced model was specifically selected as a representative paradigm to elucidate the underlying molecular mechanisms.

**Results and conclusion:**

This study successfully synthesized spherical CrCi-CDs rich in surface functional groups (hydroxyl, amino, and carboxyl groups), which exhibited excellent dispersibility in aqueous solutions. *In vivo* evaluations using PTZ, PILO, and PNC models demonstrated that CrCi-CDs significantly reduced the severity of epileptic seizures and attenuated seizure-induced spatial learning and memory deficits. Brain histopathology revealed that CrCi-CDs treatment effectively mitigated hippocampal neuronal damage. Mechanistically, CrCi-CDs exerted neuroprotective effects through multiple pathways: restoring homeostasis by correcting pathological imbalances of neurotransmitters (Glu/GABA), alleviating oxidative stress (SOD/MDA), and suppressing proinflammatory cytokine release (TNF-α, IL-1β, IL-6, IL-18). Further investigations suggest these effects may be mediated by regulating glutamate transporters, inhibiting the NF-κB inflammatory cascade, and modulating neuronal apoptosis pathways. This study confirms that bio-derived CrCi-CDs exhibit potent antiepileptic and neuroprotective properties. By simultaneously targeting neurotransmission, inflammation, and oxidative stress, CrCi-CDs emerge as a highly promising therapeutic candidate with a favorable safety profile, providing a scientific rationale for the development of biomass-derived nanomedicines in epilepsy management.

## 1 Introduction

Epilepsy constitutes a persistent neurological disorder defined by spontaneous, repetitive manifestations of central nervous system dysfunction, currently affecting approximately 50 million individuals worldwide (Beghi et al., 2015; Thijs et al., 2019). Despite the availability of symptomatic treatments, nearly 80% of patients, particularly in resource-limited settings, remain poorly managed (Yu et al., 2019). The pathogenesis of epilepsy is primarily driven by the disruption of the excitatory/inhibitory balance, involving dysregulated neurotransmitter signaling and synaptic plasticity (Falco-Walter, 2020; Neri et al., 2022; Vezzani et al., 2016). Specifically, an aberrant surge in glutamate coupled with GABAergic dysfunction leads to severe neuronal hyperexcitability and excitotoxicity (Sánchez Fernández and Loddenkemper, 2014; Devinsky et al., 2013; Kang, 2017). This hyperexcitability is inextricably linked to oxidative stress and inflammation. Epilepsy induces a rapid accumulation of reactive oxygen species (ROS) and the release of pro-inflammatory cytokines. These factors act synergistically to damage neuronal membranes and perpetuate a pro-epileptogenic environment, ultimately complicating treatment efforts and promoting pharmacoresistance (Alyu and Dikmen, 2017; Takeuchi et al., 2006; Levin and Godukhin, 2017).

The mainstay of epilepsy treatment remains antiepileptic drugs (AEDs), which target various epileptogenic mechanisms (Pong et al., 2023; Manford, 2017; Kanner and Bicchi, 2022; Hakami, 2021). However, clinical outcomes are often far from ideal. Long-term AED use is associated with severe adverse effects, including hepatorenal toxicity, while a substantial proportion of patients fail to achieve seizure freedom (Ben Mahmoud et al., 2017). These challenges highlight the necessity for safer, more effective therapeutic agents. In recent years, carbon dots (CDs) have attracted considerable interest in the biomedical field (Kang et al., 2019; Rocco et al., 2023; Szczepankowska et al., 2023; Zhu et al., 2022). Unlike traditional quantum dots, CDs offer superior biocompatibility and ease of surface functionalization, making them suitable for medical applications. Notably, our group has reported that CDs generated during the preparation of traditional herbal medicines possess intrinsic biological activities. These bio-derived nanostructures have shown promise in regulating immune responses and exerting anti-inflammatory effects, offering a potential alternative to conventional small-molecule drugs (Zhang et al., 2018; Cheng et al., 2019; Wang et al., 2019; Zhang et al., 2020; Zhao et al., 2021; Lu et al., 2021; Chen et al., 2022; Wang et al., 2023; Cui et al., 2023).

Crinis Carbonisatus (CrCi), produced by the carbonization of human hair, is a distinctive medicine in Traditional Chinese Medicine. It has been administered clinically for millennia, primarily for its hemostatic properties and ability to resolve blood stasis. Building on this historical application, we previously isolated and characterized Carbon Dots from CrCi (CrCi-CDs), demonstrating their robust neuroprotective capacity in models of ischemic stroke (Zhang et al., 2021). Given that stroke and epilepsy share overlapping pathological cascades, specifically oxidative stress, excitotoxicity, and inflammatory damage. We hypothesized that CrCi-CDs could offer broad-spectrum protection for the central nervous system. Consequently, the ability of CrCi-CDs to modulate cerebral neurotransmission, attenuate neuroinflammation, and suppress neuronal apoptosis substantiates their promising therapeutic potential as antiepileptic agents.

This research utilized a trio of acute epilepsy models to assess the capacity of CrCi-CDs to suppress inflammatory responses and alleviate oxidative injury. The study integrated behavioral testing (seizure severity scoring, spatial exploration and learning ability bssessment) with histopathological examination. Furthermore, molecular investigations focused on elucidating the roles of neurotransmitter imbalance, inflammation, and apoptotic pathways in the observed neuroprotection.

## 2 Results

### 2.1 Characterization of CrCi-CDs

**FIGURE 1:**
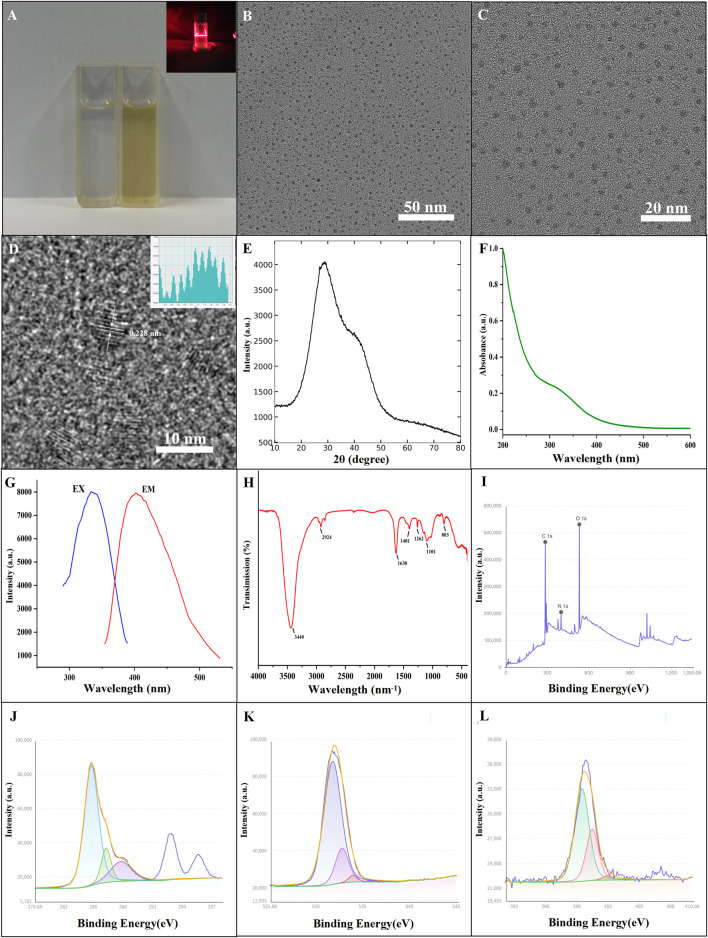
Characterization of CrCi-CDs. **(A)** Photographs of aqueous solutions and the Tyndall effect (inset). **(B,C)** TEM images at different magnifications. **(D)** HRTEM image showing lattice spacing. **(E)** XRD pattern. **(F)** UV-Vis absorption spectrum. **(G)** Fluorescence emission spectrum. **(H)** FTIR spectrum. **(I)** XPS survey scan. **(J–L)** High-resolution XPS spectra of C 1s, O 1s, and N 1s.

Visual inspection (Figure 1A) shows a pale yellow solution displaying the Tyndall phenomenon, characteristic of colloidal systems. Morphological analysis via TEM (Figures 1B,C) confirms that the CrCi-CDs are well-dispersed nanospheres with uniform dimensions. High-resolution TEM (HRTEM) analysis (Figure 1D) provided insights into the atomic arrangement of the nanodots, revealing well-defined lattice fringes. The measured interlayer d-spacing of 0.228 nm is in excellent agreement with the characteristic lattice parameters of graphitic carbon, confirming the crystalline nature of the CrCi-CDs.

As shown in Figure 1E, the amorphous nature of the CrCi-CDs was evidenced by an XRD diffraction peak centered at 2θ = 29.399°. Optical characterization (Figure 1F) revealed a smooth UV-Vis absorption curve with a weak shoulder around 320 nm, attributed to n-π* (C=O) or π-π* (C=C) transitions. Fluorescence analysis indicated that the CrCi-CDs emit bright blue light under a 365 nm UV lamp, with a peak emission at 402 nm when excited at 334 nm (Figure 1G).

Surface functionalization was corroborated by FTIR (Figure 1H) and XPS analyses. The FTIR spectrum displayed characteristic peaks for O-H/N-H (3,440 cm^−1^), C-H (2,924 cm^−1^), and C=O (1,630 cm^−1^) groups. Further peaks at 1,401 cm^−1^ (C-N/N-H/C-H), 1,262 cm^−1^ (C-OH), 1,101 cm^−1^ (C-O-C), and 863 cm^−1^ (aromatic C-H) suggest a surface rich in hydroxyl, amino, and carboxylate moieties.

Elemental composition was quantified via XPS survey scans (Figure 1I), yielding 70.7% C, 6.13% N, and 23.17% O at binding energies of 285.08, 400.08, and 532.08 eV, respectively. High-resolution spectra (Figures 1J–L) further resolved the bonding states: C1s deconvolution showed C-C, C-N, C-O, and C=O species; O1s fitting revealed C-O (531.0 eV) and C=O (532.0 eV) bonds; and N1s analysis confirmed the presence of C-N (399.5 eV) and C=N (400.5 eV) structures. These results align with prior data, confirming the successful and stable preparation of CrCi-CDs.

### 2.2 Evaluation of epilepsy inhibition and memory restoration by CrCi-CDs

**FIGURE 2:**
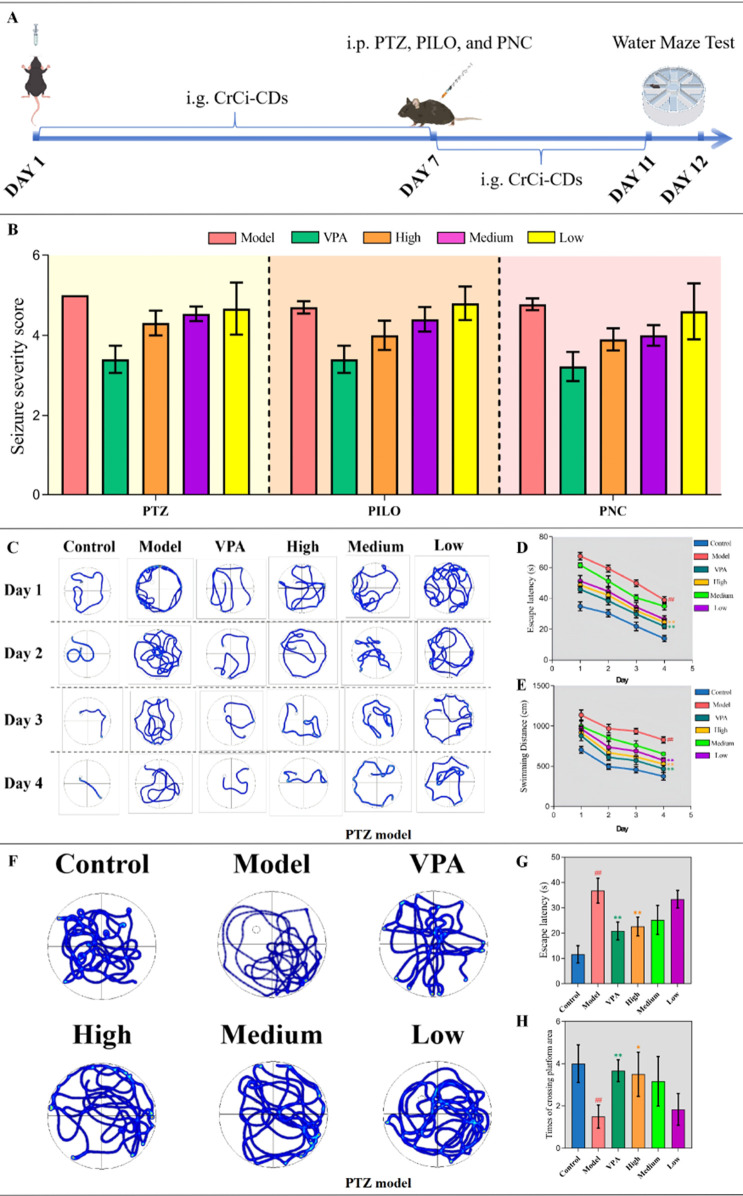
CrCi-CDs attenuate epilepsy severity and cognitive deficits. **(A)** Experimental design and dosing regimen. **(B)** Evaluation of epilepsy severity in PTZ, PILO, and PNC models. **(C–H)** Morris water maze experiment in PTZ model: representative trajectories, escape latency, and swimming distance during the navigation phase **(C–E)**; trajectories, latency, and platform crossings during the probe phase **(F–H)**. Data are presented as mean ± SD (n = 7). ^##^ *P* < 0.01 vs. control group. ^**^ *P* < 0.01 and ^*^ *P* < 0.05 vs. model group.

As illustrated in the experimental timeline (Figure 2A), mice received oral CrCi-CDs for 7 days prior to epilepsy induction. Figure 2B demonstrates that CrCi-CDs intervention reduced epilepsy severity across all three epilepsy models. Notably, the high-dose CrCi-CDs and positive control groups (VPA) exhibited the most significant alleviation of epilepsy symptoms.

To evaluate spatial learning and memory, the PTZ model was subjected to the Morris water maze. Representative swimming trajectories (Figure 2C) revealed disorganized search patterns in the PTZ model group, which were regularized by drug intervention. Quantitatively, the PTZ model group exhibited significantly prolonged escape latency and increased swimming distances compared to controls (*P* < 0.01, Figures 2D,E). However, intervention with VPA and high-dose CrCi-CDs significantly mitigated these learning deficits from day 1 to day 4 (*P* < 0.01).

In the spatial exploration test on day 5 (Figure 2F), the PTZ model mice showed poor memory retention, predominantly searching quadrants opposite the target. Statistical analysis confirmed that the PTZ model group had significantly increased latency and fewer platform crossings compared to controls (*P* < 0.01, Figures 2G,H). Conversely, VPA and high-dose CrCi-CDs effectively reversed these impairments, significantly reducing latency (*P* < 0.01) and increasing platform crossings (*P* < 0.05 and *P* < 0.01, respectively), demonstrating the restoration of spatial memory.

### 2.3 Effects of CrCi-CDs on brain tissue pathology in epileptic mice

**FIGURE 3:**
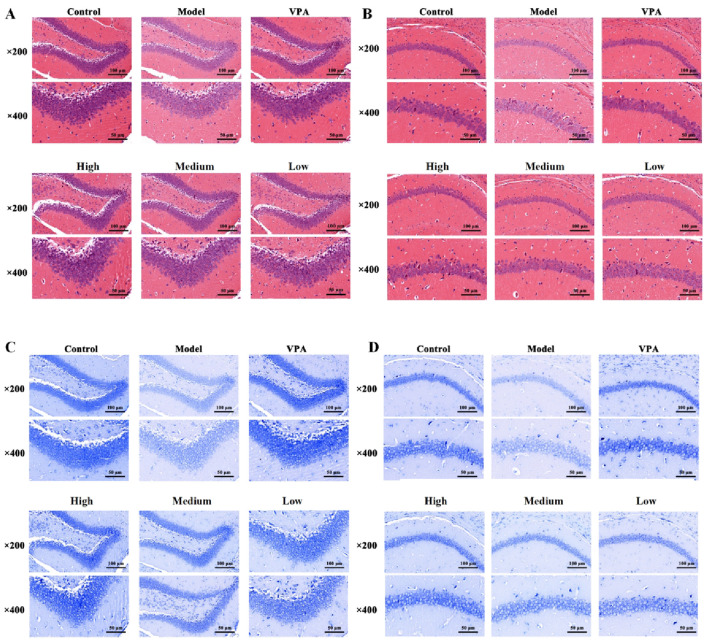
Effects of CrCi-CDs on the pathological morphology of the DG (Dentate Gyrus) and CA1 (Cornu Ammonis 1) region of the hippocampus in the brain tissue of epileptic mice induced by PTZ. **(A,B)** HE staining of the DG **(A)** and CA1 **(B)** region. **(C,D)** Nissl staining of the DG **(C)** and CA1 **(D)** region.

**FIGURE 4:**
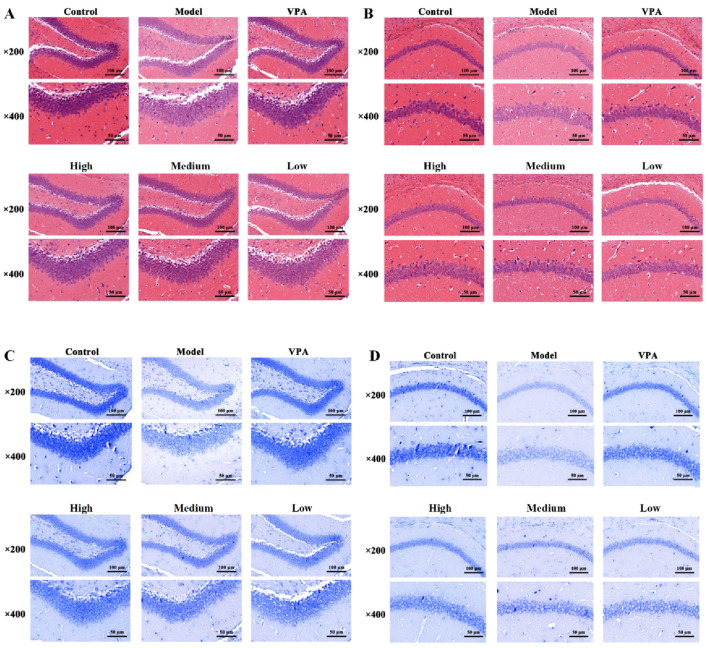
Effects of CrCi-CDs on the pathological morphology of the DG and CA1 region of the hippocampus in the brain tissue of epileptic mice induced by PILO. **(A,B)** HE staining of the DG **(A)** and CA1 **(B)** region. **(C,D)** Nissl staining of the DG **(C)** and CA1 **(D)** region.

**FIGURE 5:**
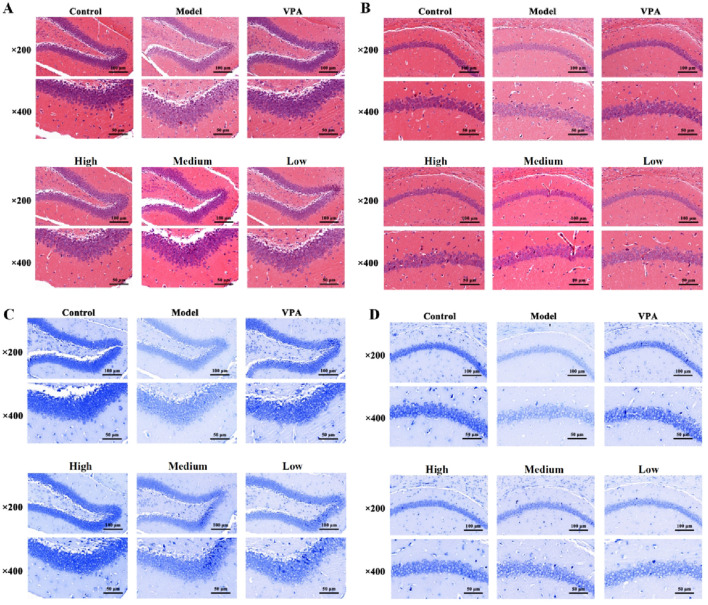
Effects of CrCi-CDs on the pathological morphology of the DG and CA1 region of the hippocampus in the brain tissue of epileptic mice induced by PNC. **(A,B)** HE staining of the DG **(A)** and CA1 **(B)** region. **(C,D)** Nissl staining of the DG **(C)** and CA1 **(D)** region.

Histological analysis of the PTZ-induced model (Figures 3A,B) revealed severe structural disarray in hippocampal tissue compared to controls. The PTZ model group was characterized by widened intercellular spaces, cellular swelling, cytoplasmic vacuolization, and evident neuronal necrosis. In contrast, these morphological defects were markedly alleviated following the administration of VPA and various concentrations of CrCi-CDs. Furthermore, Nissl staining (Figures 3C,D) demonstrated that PTZ administration caused a marked depletion of cytoplasmic Nissl bodies, a defect that was effectively reversed by CrCi-CDs and VPA treatment. Consistent neuroprotective effects were observed in the PILO and PNC epilepsy models (Figures 4, 5), confirming that CrCi-CDs possess efficacy in mitigating neuronal damage across different epilepsy paradigms.

### 2.4 CrCi-CDs attenuate neuroinflammation and mitigate neural tissue injury

**FIGURE 6:**
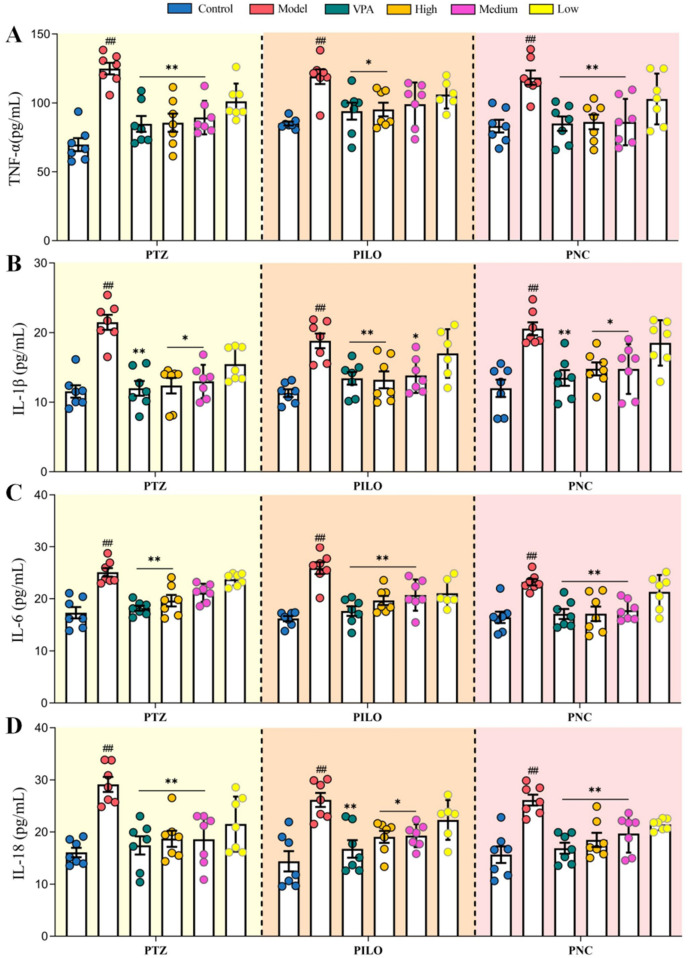
Impact of CrCi-CDs on cerebral inflammatory factors across PTZ, PILO, and PNC induced epilepsy models. **(A)** TNF-α levels, **(B)** IL-1β levels, **(C)** IL-6 levels, **(D)** IL-18 levels. Data are presented as mean ± SD (n = 7). ^##^ *P* < 0.01 vs. control group. **P < 0.01 and *P < 0.05 vs. model group.

The massive release of cytokines by glial cells and neurons triggers inflammatory cascades that are known to lower the seizure threshold and aggravate neurotoxicity (Rana and Musto, 2018). In this study, we specifically focused on the expression profiles of TNF-α, IL-1β, IL-6, and IL-18 (Figures 6A–D). Our results revealed that regardless of the chemical convulsant used (PTZ, PILO, or PNC), the induction of epilepsy was invariably accompanied by a sharp upregulation of these pro-inflammatory markers (*P* < 0.01). This consistent spike across different models suggests a common inflammatory pathway underlying chemically induced seizures. Crucially, CrCi-CDs treatment interrupted this pathological cycle. Following administration, we observed a progressive, dose-dependent downregulation of all four cytokines. The high-dose CrCi-CDs group, in particular, exhibited a potent anti-inflammatory capacity, effectively suppressing the overexpression of TNF-α, IL-1β, IL-6, and IL-18. This data suggests that CrCi-CDs do not merely mitigate symptoms but actively improve the microenvironment of neural tissue by resolving acute neuroinflammation.

### 2.5 CrCi-CDs alleviate oxidative stress and restore the Glu/GABA balance

**FIGURE 7:**
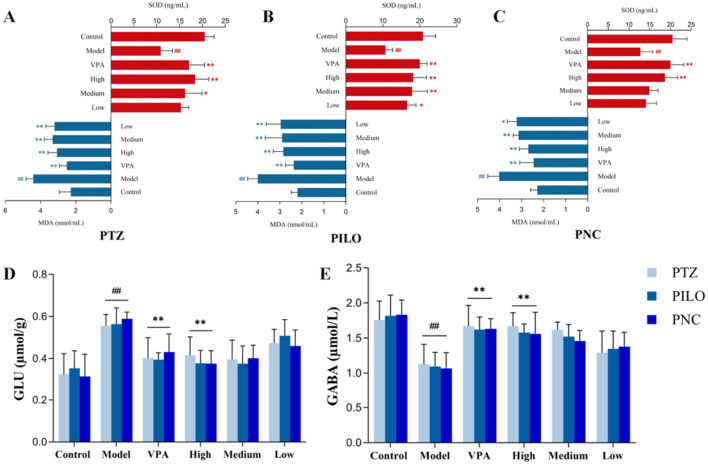
Regulation of cerebral oxidative stress and neurotransmitter by CrCi-CDs in three epilepsy models. **(A–C)** Levels of SOD and MDA in the brain tissue of PTZ- **(A)**, PILO- **(B)**, and PNC-induced **(C)** models. **(D,E)** Brain concentrations of Glu **(D)** and GABA **(E)** across the three epilepsy models. Data are presented as mean ± SD (n = 7). ^##^ *P* < 0.01 vs. control group. **P < 0.01 and *P < 0.05 vs. model group.

Excessive reactive oxygen species (ROS) exacerbate epileptic brain injury by depleting antioxidant systems (e.g., SOD) and generating toxic intermediates like MDA, which serves as a reliable marker for oxidative damage (Sun et al., 2022; Wang et al., 2021). As shown in Figures 7A–C, across the three epilepsy models, the model groups exhibited significantly reduced SOD activity and elevated MDA levels compared to controls (*P* < 0.01). However, intervention with VPA and CrCi-CDs effectively normalized these markers. Specifically, VPA and high-dose CrCi-CDs significantly reversed the oxidative stress indices (*P* < 0.01), with the high-dose group demonstrating the most potent antioxidative efficacy among the CrCi-CDs treatments.

The disruption of the excitatory/inhibitory balance, characterized by dysregulated Glu and GABA expression, is a central mechanism in epilepsy. As illustrated in Figures 7D,E, the model groups in all three induction protocols showed significantly upregulated Glu and downregulated GABA levels compared to controls (*P* < 0.01). CrCi-CDs intervention dose-dependently corrected this imbalance. Notably, both VPA and high-dose CrCi-CDs significantly decreased Glu and increased GABA levels (*P* < 0.01). These biochemical findings align with the behavioral improvements, confirming that CrCi-CDs effectively ameliorate the Glu/GABA imbalance and mitigate neuronal excitotoxicity.

### 2.6 Coordinate regulation of glutamate transport, inflammation, and apoptosis by CrCi-CDs in epileptic mice

**FIGURE 8:**
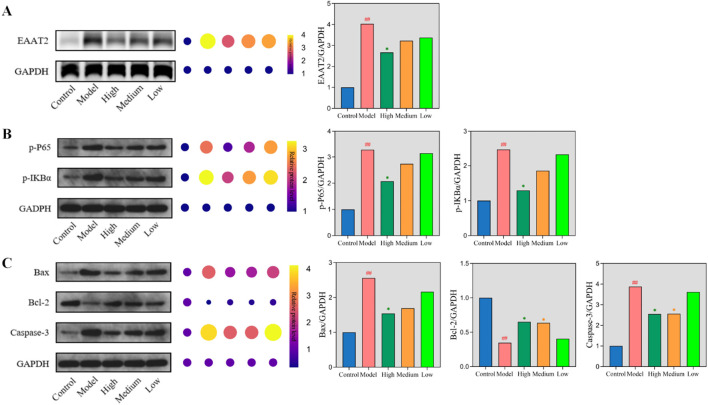
Regulation of EAAT2, NF-κB, and apoptotic pathways by CrCi-CDs in PTZ-induced epileptic mice. **(A)** EAAT2 protein levels. **(B)** Phosphorylation of IKBα and p65. **(C)** Bax, Bcl-2, and Caspase-3 expression. ##*P* < 0.01 vs. control group. **P < 0.01 and *P < 0.05 vs. model group.

Seizures induce excitotoxicity and inflammation, leading to neuronal damage (Sarlo and Holton, 2021; Vezzani et al., 2015). Validated by histological findings (Figures 3–5), we further explored the underlying mechanisms in the PTZ model by analyzing protein expression in the EAAT2, NF-κB, and apoptotic pathways. Regarding glutamate transport (Figure 8A), the model group exhibited significantly elevated EAAT2 expression compared to controls (*P* < 0.01). This likely reflects a compensatory upregulation in response to excessive synaptic glutamate. CrCi-CDs intervention downregulated EAAT2 expression, with the high-dose group showing a significant reduction (*P* < 0.05), indicating that CrCi-CDs effectively mitigated synaptic glutamate accumulation and neuronal excitability. We further assessed the NF-κB inflammatory pathway (Figure 8B). The model group showed significantly increased levels of p-IKBα and p-p65 (*P* < 0.01), indicating pathway activation. High-dose CrCi-CDs markedly decreased these phosphorylation levels (*P* < 0.05), thereby suppressing NF-κB signaling and the downstream inflammatory cascade. Finally, we evaluated neuronal apoptosis (Figure 8C). The model group displayed significantly elevated pro-apoptotic markers (Bax, Cleaved-caspase-3) and reduced anti-apoptotic Bcl-2 (*P* < 0.01). CrCi-CDs treatment significantly reversed these trends (*P* < 0.05), upregulating Bcl-2 while downregulating Bax and Caspase-3. These results confirm that CrCi-CDs exert neuroprotective effects by inhibiting epilepsy-induced neuronal apoptosis.

## 3 Discussion

Building on the millennial use of Crinis Carbonisatus (CrCi) in Traditional Chinese Medicine for epilepsy treatment and our previous identification of CrCi-CDs as bioactive nanostructures, this study investigated their antiepileptic potential. Given that epilepsy and ischemic stroke share key pathogenic mechanisms—specifically excitotoxicity and neuroinflammation—we hypothesized that CrCi-CDs would exert neuroprotective effects in epilepsy models similar to those observed in ischemia.

To comprehensively evaluate this hypothesis, we employed three acute epilepsy models (PTZ, PILO, and PNC) (Löscher, 2017). Despite distinct initiation mechanisms, these agents converge on a critical downstream pathology: the elevation of synaptic glutamate levels (Akyuz et al., 2021; Zenki et al., 2018; Gröticke et al., 2007; Pal et al., 2000; Zhu et al., 2018). This glutamate surge drives neuronal hyperexcitability, oxidative stress, and apoptosis. Our results demonstrated that CrCi-CDs administration effectively interrupted this pathological process. CrCi-CDs dose-dependently reduced seizure severity across all models and ameliorated cognitive deficits in spatial learning and memory, particularly in the PTZ-induced epilepsy model. These consistent findings support the broad-spectrum anticonvulsant properties of CrCi-CDs.

Mechanistically, the neuroprotective effects appear to be mediated through the regulation of neurotransmission and cellular stress responses. CrCi-CDs rectified the imbalance between excitatory (Glu) and inhibitory (GABA) signaling, thereby attenuating excitotoxicity (Sears and Hewett, 2021; Niquet et al., 2023). Concurrently, the treatment suppressed pro-inflammatory cytokines and mitigated oxidative stress. These biochemical improvements align with previous studies on biomass-derived nanomaterials (Wang et al., 2023; Lu et al., 2021; Humaera et al., 2021), highlighting the antioxidant capacity of CrCi-CDs, presumably attributed to their surface functional groups.

To elucidate the regulation of glutamate homeostasis, we selected the PTZ model as a representative paradigm to examine EAAT2 expression (Takahashi et al., 2015; Vandenberg and Ryan, 2013; Bjørnsen et al., 2014). While EAAT2 dysfunction is often linked to neurological disorders (Zhang et al., 2025; Bacigaluppi et al., 2016; Chung et al., 2008), our specific PTZ-induced paradigm revealed a compensatory upregulation of EAAT2 in the model group, likely an adaptive response to accelerate glutamate clearance (Sugimoto et al., 2018; Petr et al., 2015; Peterson and Binder, 2019). Notably, CrCi-CDs treatment normalized EAAT2 levels towards the baseline (Figure 8A). We interpret this observation not as a suppression of transporter function, but rather as a restoration of homeostasis. By reducing upstream glutamate release and dampening excitability, the treatment likely alleviates the physiological burden on astrocytes, removing the stimulus for compensatory overexpression. However, we strictly acknowledge a limitation here: as this study relied on Western blot analysis, functional glutamate uptake assays were not performed. Therefore, the precise functional kinetics of glutamate transport following CrCi-CDs treatment warrant further investigation.

**FIGURE 9:**
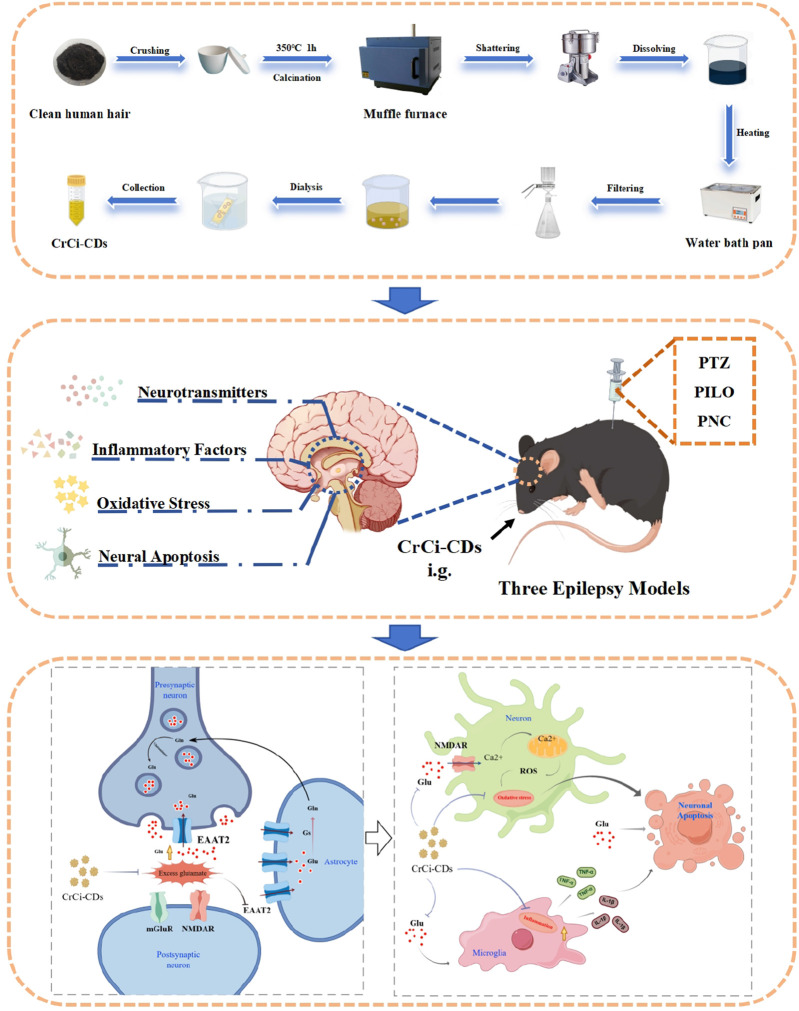
Schematic diagram of the preparation process and antiepileptic mechanism of CrCi-CDs. Drawn by By Figdraw.

Furthermore, we investigated the NF-κB signaling pathway to explore the anti-inflammatory mechanism (Brigelius-Flohé and Flohé, 2011; Cai and Lin, 2022). Western blot analysis revealed that CrCi-CDs treatment effectively inhibited the phosphorylation of IκBα and p65. By blocking this activation step (Lee et al., 2013), CrCi-CDs prevented the nuclear translocation of NF-κB, thereby suppressing the downstream transcription of pro-inflammatory cytokines. Regarding neuronal survival, the intervention modulated the Bcl-2 family signaling (Zhang et al., 2019; Martinou and Youle, 2011; Gross, 2016; Asadi et al., 2022). As shown in Figure 9C, CrCi-CDs suppressed pro-apoptotic markers (Bax, Caspase-3) and elevated anti-apoptotic Bcl-2 levels, shifting the cellular machinery towards survival and providing protection against epilepsy-induced degeneration.

It is worth noting that while the detailed molecular verifications in this study were conducted specifically in the PTZ model, the observed neuroprotective effects are likely applicable to the PILO and PNC models. As demonstrated by our ELISA and behavioral data, CrCi-CDs exerted consistent therapeutic effects across all three models. Since PTZ, PILO, and PNC induced-seizures all converge on the common pathological downstream of glutamate excitotoxicity and neuroinflammation (Feng et al., 2005; Costa et al., 2004; Moriyama et al., 2025), it is plausible that CrCi-CDs target these shared pathways. Future studies may further validate these specific molecular targets in diverse epilepsy models to reinforce this generalization.

In summary, CrCi-CDs exert neuroprotection via a multi-pronged mechanism that involves restoring the excitatory/inhibitory neurotransmitter balance, dampening neuroinflammatory cascades, and blocking neuronal apoptosis. Crucially, unlike conventional AEDs which are frequently plagued by hepatorenal toxicity, CrCi-CDs exhibit a favorable safety profile. This hypothesis is supported by our previous experimental evidence showing negligible cytotoxicity even at high concentrations (Zhang et al., 2021), as well as the millennium-long clinical safety record of their precursor, Crinis Carbonisatus, in Traditional Chinese Medicine.

However, we strictly acknowledge a limitation of the current study: the specific *in vivo* biodistribution and long-term accumulation of CrCi-CDs were not directly assessed. Therefore, while our current findings confirm CrCi-CDs as a promising, low-toxicity adjunctive therapeutic candidate, subsequent research is strictly required to systematically map their biodistribution and metabolic clearance pathways to definitively establish their clinical safety profile.

## 4 Conclusion

In conclusion, this study successfully synthesized CrCi-CDs using human hair as the raw material. Their broad-spectrum antiepileptic potential was validated across three distinct acute epilepsy models (PTZ, PILO, and PNC), demonstrating consistent efficacy in reducing seizure severity and preserving cognitive function. Mechanistic investigations, conducted using the PTZ model as a representative paradigm, revealed that CrCi-CDs exert neuroprotection via a multi-pronged strategy. Specifically, the treatment normalized epilepsy-induced compensatory EAAT2 expression by reducing synaptic glutamate load, rather than direct inhibition. Concurrently, CrCi-CDs suppressed neuroinflammation by blocking the NF-κB signaling pathway (evidenced by reduced phosphorylation of IκBα and p65), attenuated oxidative stress, and mitigated neuronal apoptosis by modulating the Bcl-2/Bax/Caspase-3 axis. Crucially, unlike conventional AEDs which are frequently associated with hepatorenal toxicity, CrCi-CDs exhibit a favorable safety profile. This is substantiated by our previous experimental evidence showing negligible cytotoxicity even at high concentrations, as well as the millennium-long clinical safety record of their precursor, Crinis Carbonisatus, in Traditional Chinese Medicine. Collectively, these findings confirm that CrCi-CDs effectively target the core pathological triad of epilepsy—excitotoxicity, inflammation, and oxidative stress. By demonstrating potent neuroprotection with high biocompatibility, this study lays a solid foundation for the potential integration of CrCi-CDs into modern antiepileptic regimens.

## 5 Materials and methods

### 5.1 Chemicals

The study utilized PTZ, VPA and sodium pentobarbital from Sigma-Aldrich (United States) and PILO from APExBIO Technology (United States). Domestically, PNC and 1,000 Da dialysis membranes were attained from North China Pharmaceutical Co., Ltd. and Beijing Ruida Henghui Technology Development Co., Ltd., respectively. All experimental solutions were prepared using deionized water (DW) to ensure consistency across the study.

### 5.2 Preparation of CrCi-CDs

CrCi-CDs were fabricated from human hair in accordance with our previously described methodology (Zhang et al., 2021). Briefly, cleaned hair was desiccated at 60 °C for 24 h prior to being placed in crucibles. The samples underwent a specific thermal treatment: a 20-min temperature ramp to 70 °C, followed by carbonization at 350 °C for 1 h. Upon cooling to room temperature, the carbonized residue was ground into a fine powder and suspended in deionized water (DW). The suspension was subsequently boiled at 100 °C for 1 h to extract the CDs. To purify the product, the mixture was filtered through a 0.22 µm membrane, and the filtrate was dialyzed against DW for 72 h (MWCO: 1,000 Da) to obtain the final CrCi-CDs solution. Figure 9 briefly illustrates the preparation process of CrCi-CDs.

### 5.3 Characterization of CrCi-CDs

Spectrofluorimetric data were acquired with a Shimadzu RF5-5301 PC instrument, and UV-visible profiles were obtained through a TU-1991 spectrophotometer. Functional group composition was monitored via FTIR (Nicolet 6700) across a spectral range of 500–4,000 cm^−1^. To evaluate the physical dimensions and structural features, TEM images were captured using a JEM-2100F microscope (operating at 200 kV). These analyses provided comprehensive insights into the optical and structural attributes of the resultant carbon dots.

### 5.4 Experimental procedure

Male C57BL/6 mice (20.0 ± 2.0 g) were acclimatized under standard laboratory conditions: a controlled temperature of 24.0 °C ± 1.0 °C, 55%–65% relative humidity, and a 12-h light/dark cycle. Standard chow and water were provided *ad libitum*.

The animals were stratified into three independent batches to establish different epilepsy models (Three batches, 42 mice in each batch). Within each batch, mice were randomized into six groups (n = 7): Control, Model, positive control (VPA, 200 mg/kg), and three CrCi-CDs treatment groups (low dose group: CrCi-CDs 1.5 mg/kg; medium dose group: CrCi-CDs 3 mg/kg; high dose group: CrCi-CDs 6 mg/kg). The dosing regimen for CrCi-CDs was selected based on our previous study demonstrating neuroprotection in ischemic stroke (Zhang et al., 2021), combined with the conversion of the standard clinical dosage of Crinis Carbonisatus in Traditional Chinese Medicine to murine equivalents. All treatments were administered via intragastric gavage once daily for seven consecutive days, while the Control and Model groups received an equivalent volume of saline.

On Day 7, 60 min after the final prophylactic treatment, epilepsy were induced via intraperitoneal (i.p.) injection. The three cohorts were challenged with PTZ (65 mg/kg), PILO (280 mg/kg), or PNC (4.2 g/kg), respectively (Janisset et al., 2022; Curia et al., 2008; Akdogan et al., 2008). All control groups received i.p. saline injections. Animals in each group were monitored continuously for 30 min post-injection to observe the severity of epilepsy and quantified according to the Racine scale (Racine, 1972).

### 5.5 Morris water maze test

The PTZ-induced epilepsy model, a classic paradigm in epilepsy research, was selected for this assay due to its high value in primary drug screening and mechanistic investigation. Since PTZ-induced seizures typically result in hippocampal excitotoxicity and subsequent cognitive impairment, the Morris water maze was employed to evaluate the preventive neuroprotective efficacy of CrCi-CDs against these seizure-associated cognitive deficits.

A circular pool with dimensions of 90 cm (diameter) by 50 cm (height) was employed for the water maze tests. The water temperature was stabilized at 22 °C–24 °C, and all experimental data were recorded using an EthoVision XT automated monitoring system. The pool was divided into four quadrants, with visual cues mounted on the walls to facilitate spatial orientation. A hidden escape platform (9 cm diameter) was submerged 1 cm below the water surface in the target quadrant. lighting was kept soft and diffuse to prevent reflections.

#### 5.5.1 Spatial learning test

The test was conducted over four consecutive days starting at 9:00 a.m. daily. Mice underwent four trials per day, starting from distinct randomized quadrants with their heads facing the pool wall. The time taken to locate the platform (escape latency) was recorded with a 90-s cutoff. Unsuccessful trials concluded with the researcher guiding the mouse to the platform for a 30 s stay. To avoid hypothermia, mice were thoroughly dried without delay after each exit from the water, ensuring they remained warm and dry before returning to their home cages.

#### 5.5.2 Spatial memory test

On Day 5, a spatial exploration test was performed to assess memory retention. The platform was removed, and mice were introduced from the quadrant opposite the target zone. The swimming trajectory was tracked for 60 s, and the number of platform crossings was quantified.

### 5.6 Sample collection

Following the behavioral test, after a 24-h recovery period, mice in each group were anesthetized via intraperitoneal injection of sodium pentobarbital (40 mg/kg). Blood samples were then rapidly collected through the retroorbital plexus. Mice were then humanely euthanized by decapitation under anesthesia. Whole brains were rapidly harvested on ice, rinsed with pre-chilled saline to remove residual blood, blotted dry, and weighed. The brain tissue was then bisected along the sagittal midline. The left hemisphere was snap-frozen in liquid nitrogen and stored at −80 °C for biochemical analysis, while the right hemisphere was fixed in 4% paraformaldehyde for histopathological examination.

### 5.7 Histological section staining

Fixed brain tissues were dehydrated through a graded ethanol series (50%–100%), cleared in xylene, and embedded in paraffin wax. Coronal sections (3 μm thick) were cut using a microtome. Prior to staining, all sections underwent standard deparaffinization and rehydration through a series of xylene and graded alcohols.

Hematoxylin and Eosin (H&E) Staining: Rehydrated sections were stained with hematoxylin for 5 min, differentiated in warm water for 10 min, and counterstained with eosin for 2 min. After dehydration and mounting with neutral resin, general neuronal morphology was examined under a light microscope.

Nissl Staining: Sections were incubated in toluidine blue solution at 37 °C for 4 h. Following rinsing and dehydration, the slides were cleared in xylene and mounted. The integrity of Nissl bodies in the hippocampus was evaluated microscopically to assess neuronal damage.

### 5.8 Analysis of relevant cytokines

After mechanically dissociating the frozen tissue in a pre-chilled environment, the brain homogenate was centrifuged to obtain the supernatant. The concentrations of amino acid neurotransmitters (GABA, Glu) and inflammatory cytokines (TNF-α, IL-1β, IL-6, IL-18) were quantified using specific ELISA kits purchased from Beijing Dongge Boye Biotechnology Co., Ltd. (Beijing, China). All procedures were conducted strictly according to the manufacturer’s instructions, and optical density was measured at 450 nm. Simultaneously, oxidative stress markers were assessed using biochemical assay kits from the same manufacturer. Specifically, Superoxide Dismutase (SOD) activity was determined via the WST-8 method (Ukeda et al., 1999), while Malondialdehyde (MDA) levels were measured using the Thiobarbituric Acid (TBA) method (Ohkawa et al., 1979). Protein concentrations were determined using the Bradford assay to normalize the data.

### 5.9 Western blot analysis

Brain tissues were lysed in RIPA buffer containing protease inhibitors, and the resulting protein concentration was measured using a BCA kit (Bradford, 1976). Equal protein quantities were fractionated on SDS-PAGE gels and transferred to polyvinylidene fluoride (PVDF) membranes. After 1.5 h of blocking with 5% skim milk powder, the membrane was incubated overnight with the primary antibody on a rocking incubator, followed by incubation with the secondary antibody at room temperature for 1 h. Following TBST rinses, target protein signals were detected via enhanced chemiluminescence and quantified using a digital imaging analysis system.

### 5.10 Statistical analysis

Data are expressed as mean ± standard deviation (SD). Statistical comparisons were performed using GraphPad Prism software (Version 9.0). For comparisons among multiple groups, one-way analysis of variance (ANOVA) was performed, followed by Tukey’s multiple comparisons test. For non-normally distributed data (e.g., behavioral seizure scores), the Kruskal–Wallis test followed by Dunn’s multiple comparisons test was applied. A *P* < 0.05 was considered statistically significant. The criteria for significance were defined as *P < 0.05, **P < 0.01, and ***P < 0.001 vs. the corresponding control or model group as indicated in each figure and table. For *in vivo* animal experiments, each group consisted of seven biological replicates (n = 7).

## Data Availability

The original contributions presented in the study are included in the article/supplementary material, further inquiries can be directed to the corresponding authors.
